# Comparison of PD-L1 Expression Between Preoperative Biopsy Specimens and Surgical Specimens in Non-Small Cell Lung Cancer †

**DOI:** 10.3390/cancers17030398

**Published:** 2025-01-25

**Authors:** Tadashi Sakaguchi, Akemi Iketani, Kentaro Ito, Yoichi Nishii, Koji Katsuta, Osamu Hataji

**Affiliations:** 1 Department of Respiratory Medicine, Matsusaka Municipal Hospital, 1550 Tonomachi, Matsusaka 515-0073, Mie, Japan; 2 Pathology Department, Matsusaka Municipal Hospital, 1550 Tonomachi, Matsusaka 515-0073, Mie, Japanmchpathc@quartz.ocn.ne.jp (K.K.)

**Keywords:** programmed death ligand-1 expression, non-small cell lung cancer, biopsy specimens, surgical specimens

## Abstract

Background: Recent advances in perioperative immunotherapies have led to a new era in the perioperative treatment of resectable, non-small cell lung cancer (NSCLC). Although the choice of neoadjuvant, adjuvant or perioperative immunotherapy remains controversial, few reports have compared programmed death ligand-1 (PD-L1) expression as a biomarker between preoperative biopsy specimens and surgical specimens. Methods: We retrospectively reviewed consecutive patients with NSCLC whose preoperative biopsy specimens and surgical specimens were tested for PD-L1 (22C3) and PD-L1 (SP263), respectively, from June 2022 to February 2024. The three categorical classifications of PD-L1 expression (negative [<1%], low [1–49%], and high [≥50%]) were compared between the two tests. Results: Of the 33 patients, 13 patients had negative PD-L1 expression, 9 patients had low PD-L1 expression and 11 patients had high PD-L1 expression with preoperative biopsy specimens, while 18 patients had negative PD-L1 expression, 10 patients had low PD-L1 expression and 5 patients had high PD-L1 expression with surgical specimens. The concordance rate for the three categorical classifications of PD-L1 expression between the preoperative biopsy specimens and surgical specimens was 57.6%. Conclusions: PD-L1 expression may differ between preoperative biopsy specimens and surgical specimens. PD-L1 expression evaluated using small biopsy specimens may be largely influenced by chance due to intra-tumoral heterogeneity.

## Simple Summary

Recently, perioperative immunotherapies have advanced and have become guideline recommended therapies. The timing of the implementation of immunotherapy, including neoadjuvant, adjuvant or perioperative, remains controversial due to the lack of direct comparative trials of the respective treatments. Although most studies related to perioperative immunotherapies showed a tendency for higher programmed death ligand-1 (PD-L1) expression categories to experience more pronounced benefits from the addition of immune checkpoint inhibitors to, or following, chemotherapy, few reports have compared PD-L1 expression as a biomarker between preoperative biopsy specimens and surgical specimens. This study demonstrated that the concordance of PD-L1 expression between preoperative biopsy specimens and surgical specimens was not high, and suggested that PD-L1 expression evaluated using small biopsy specimens may be largely influenced by chance due to intra-tumoral heterogeneity.

## 1. Introduction

Immune checkpoint inhibitors (ICIs), such as programmed death-1/programmed death ligand-1 (PD-1/PD-L1) inhibitors, have demonstrated remarkable clinical benefits in the treatment of metastatic and locally advanced non-small cell lung cancer (NSCLC) [1,2,3,4]. They have already become a crucial, guideline-recommended therapy for locally advanced and advanced NSCLC worldwide [5,6,7]. Their momentum shows no signs of slowing down, with recent advances in perioperative immunotherapies marking the beginning of a new era in the perioperative treatment of NSCLC [8,9,10].

PD-L1 expression in tumor cells, assessed using immunohistochemistry (IHC) assays, has evolved alongside advances in ICI therapy as a key biomarker for predicting the efficacy of PD-1/PD-L1 inhibitors in advanced NSCLC. Clinical trials have been conducted using different PD-L1 IHC assays for each PD-1/PD-L1 inhibitor, and the effectiveness of the treatments have been established with the assessment of each PD-L1 IHC assay. Therefore, each PD-1/PD-L1 inhibitor has a different companion or complementary diagnostic: Dako 28-8 with nivolumab (Bristol-Myers Squibb, Princeton, NJ, USA), Dako 22C3 with pembrolizumab (Merck & Co., Inc. Rahway, NJ, USA), Ventana SP263 with durvalumab (AstraZeneca, Cambridge, UK), and Ventana SP142 with atezolizumab (Roche, Basel, Switzerland) in locally advanced, and advanced NSCLC [11]. The Blueprint project phase 1 study has revealed that three of the four PD-L1 IHC assays (Dako 22C3, Dako 28-8, and Ventana SP263) were closely aligned on tumor cell (TC) staining whereas the Ventana SP142 assay showed consistently fewer TCs stained in the same specimens [12]. The Blueprint project phase 2 study has confirmed that three of the five currently available PD-L1 IHC assays (Dako 22C3, Dako 28-8, and Ventana SP263) show comparable staining characteristics on TCs in the same specimens, whereas the Ventana SP142 assay shows less sensitivity and the Dako 73-10 IHC assay shows higher sensitivity to detect PD-L1 expression [13].

As immunotherapies advance in the perioperative treatment of NSCLC, the IMpower010 trial, a randomized, multicenter, open-label phase III study, demonstrated that adjuvant atezolizumab treatment improved disease-free survival (DFS) compared to best supportive care (BSC) in patients with resected stage II–IIIA NSCLC with PD-L1 expression of 1% or more, as evaluated with Ventana SP263 (hazard ratio [HR] 0.66, 95% confidence interval [CI]: 0.50–0.88; *p* = 0.0039) [8]. This led to the approval of atezolizumab as adjuvant treatment following resection and platinum-based chemotherapy in patients with stage II–IIIA NSCLC with PD-L1 expression of 1% or more, and the approval of Ventana SP263 as the companion diagnostic. In the IMpower010 trial, it was demonstrated that as PD-L1 expression increased—commonly classified into three categories: <1% (negative), 1–49% (low expression), and ≥50% (high expression)—the hazard ratios (HR) for disease-free survival (DFS) and overall survival (OS) improved in the atezolizumab group accordingly compared with the BSC group [8,14]. In addition, the CheckMate 816 trial, which evaluated neoadjuvant nivolumab plus chemotherapy followed by resection, and the KEYNOTE-671 trial, which studied neoadjuvant pembrolizumab plus chemotherapy followed by resection and adjuvant pembrolizumab, both demonstrated promising treatment effects in terms of event-free survival and pathological response compared to neoadjuvant chemotherapy alone followed by surgery [9,10]. This resulted in the approval of both regimens worldwide. Furthermore, both trials also showed a tendency for higher PD-L1 expression categories to experience more pronounced benefits from the addition of ICIs to chemotherapy [9,10].

Although the choice of adjuvant, neoadjuvant, or perioperative immunotherapies remains controversial, the degree of PD-L1 expression categories is considered crucial to maximize the benefit of PD-1/PD-L1 inhibitors in perioperative settings. Few reports, however, have compared PD-L1 expression as a biomarker between preoperative biopsy specimens and surgical specimens. Therefore, we compared PD-L1 expression between preoperative biopsy specimens and surgical specimens in this study.

## 2. Materials and Methods

### 2.1. Patient Selection

This retrospective study was conducted at Matsusaka Municipal Hospital, Japan. In our routine clinical practice, Dako 22C3 PD-L1 expression is assessed at diagnosis for NSCLC of any stage, using either biopsy specimens, if appropriate, or surgical specimens, and Ventana SP263 PD-L1 expression is assessed for consideration of adjuvant atezolizumab in surgical cases without preoperative immunotherapy and in pathological stage IIA-IIIB patients. We reviewed electronic data from consecutive patients with NSCLC who were pathologically diagnosed with stage IIA-IIIB NSCLC and whose preoperative biopsy specimen and surgical specimen were clinically tested for Dako 22C3 PD-L1 expression and Ventana SP263 PD-L1 expression, respectively, from June 2022 to February 2024. Cases were excluded if either the 22C3 test on the preoperative biopsy specimens or the SP263 test on the surgical specimens were absent, or if the cases received neoadjuvant therapy. Clinical data assessments included: patient characteristics, pathological stage, sampling methods, surgical methods, pathological findings, and the results of PD-L1 expression. This study was performed in accordance with the Declaration of Helsinki. This study was approved by the institutional review board of Matsusaka Municipal Hospital (IRB number J-282-240614-5-1). Since this was a retrospective study, informed consent was not obtained. Instead, an opt-out method was employed to ensure that participants had the opportunity to withdraw from this study.

### 2.2. FFPE Sample Preparation and Immunohistochemical Analysis

As we previously reported [15], small tissue samples collected by transbronchial biopsy (TBB), transbronchial needle aspiration (TBNA), and computed tomography-guided percutaneous needle biopsy (CTNB) were immediately placed in 10% neutral buffered formalin (NBF) and fixed for about 12 to 24 h at room temperature (RT). In cases of lobectomy, we took 10 mm × 10 mm samples in tumor rich areas for genetic testing concurrently when sampling for intraoperative rapid diagnosis (IRD), and the samples were immediately placed in 10% NBF and fixed over 24 to 48 h at RT for appropriate formalin fixation. After sampling for genetic testing, 10% neutral buffered formalin (NBF) is injected into the bronchial tubes, the area surrounding the tumor, and the lung parenchyma of resected lung using a syringe. The surgical specimen was returned to the operating room and was stored in a refrigerator at 4 °C for less than 3 h. After the surgeon’s post-operative explanation, the surgical specimen was fully immersed in a large container filled with 10% NBF. The surgical specimen fixed in the container was transported to the pathology room the following day and the specimen was processed for sectioning the day after surgery. In cases where the surgical resection was performed on Friday, the duration of formalin fixation was permitted for up to 72 h, and sectioning was performed the following Monday. Formalin-fixed tissues underwent serial processing and were embedded in paraffin to create formalin-fixed and paraffin-embedded (FFPE) blocks with meticulous care to avoid nuclease contamination. For small tissue samples, a couple of samples were embedded in a FFPE block. The number of tumor cells and tumor content of the samples stained with hematoxylin and eosin were evaluated by skilled cytopathologists. Regarding PD-L1 expression testing, the specimens including at least 100 viable tumor cells were selected, and FFPE tissue sections of 5 μm thickness were submitted to LSI Medience Laboratories (Tokyo, Japan) for testing. Immunostaining using clone SP263 (Roche Diagnostics) was performed with a Ventana BenchMark platform (Ventana Medical Systems) and immunostaining using 22C3 pharmDx (Dako, Agilent Technologies) kit was performed using a Dako Autostainer Link 48 platform in the LSI Medience Laboratory in accordance with the manufacturer’s instructions. In both assays, positive and negative control slides for PD-L1 were stained alongside the specimens to confirm the accuracy of the test. A trained pathologist in the testing laboratory evaluated the tumor proportion score (TPS) for each case. Using cutoff values commonly used in clinical practice and clinical trials, we defined PD-L1 expression of <1% as negative, 1–49% as low expression, and ≥50% as high expression, and categorized each specimen into one of the three classification groups.

### 2.3. Outcomes

For the main outcome, we evaluated the concordance rate using three categorical classifications of PD-L1 expression between the 22C3 of the preoperative biopsy specimens and the SP263 of the surgical specimens in each case. For additional analysis, we evaluated the 22C3 of the surgical specimens, and assessed the concordance rate of the classification of the 22C3 between the preoperative biopsy specimens and the surgical specimens, and the concordance rate of the classification between the 22C3 and the SP263 of the surgical specimens, respectively.

### 2.4. Statistical Analysis

Descriptive statistics were used for categorical variables, presented as numbers (percentages), and for continuous variables, presented as medians (ranges). To compare the clinical concordance of the assays, the concordance rate, and Cohen’s κ value of the three categorical classifications (<1%, 1–49% and ≥50%) and two categorical classifications using each cutoff value (≥1% and ≥50%), were calculated. Scores of κ values less than 0.60 indicated weak agreement, 0.60–0.79 moderate agreement, 0.80–0.90 strong agreement, and above 0.90 indicated almost perfect agreement [16]. Statistical analyses were performed using SPSS software, version 26.0 (SPSS Inc., Chicago, IL, USA). *p*-values of less than 0.05 were considered statistically significant.

## 3. Results

### 3.1. Patient and Tumor Characteristics

A total of 33 patients were identified in this study. The characteristics of the 33 patients are shown in Table 1. The median patient age was 74 (47–89) years old, and 25 patients were former or current smokers. The histological subtypes included 27 adenocarcinomas and 6 squamous cell carcinomas. Most of the preoperative biopsy methods were TBB (85%), followed by CTNB (12%) from primary lesions, and only one patient’s biopsy was obtained from metastatic lymph node by EBUS-TBNA. All surgical methods were lobectomy.

### 3.2. PD-L1 Immunohistochemical Staining

The results of PD-L1 expressions with the 22C3 of preoperative biopsy specimens were as follows: negative, 13 cases (39.4%); low expression, 9 cases (27.3%); high expression, 11 cases (33.3%). The results of PD-L1 expressions with the SP263 of surgical specimens were as follows: negative, 18 cases (54.5%); low expression, 10 cases (30.3%); high expression, 5 cases (15.2%). The results of PD-L1 expressions with the 22C3 of surgical specimens were as follows: negative, 16 cases (48.5%); low expression, 7 cases (21.2%); high expression, 10 cases (30.3%).

### 3.3. Concordance of PD-L1 Expressions

**Figure 1:**
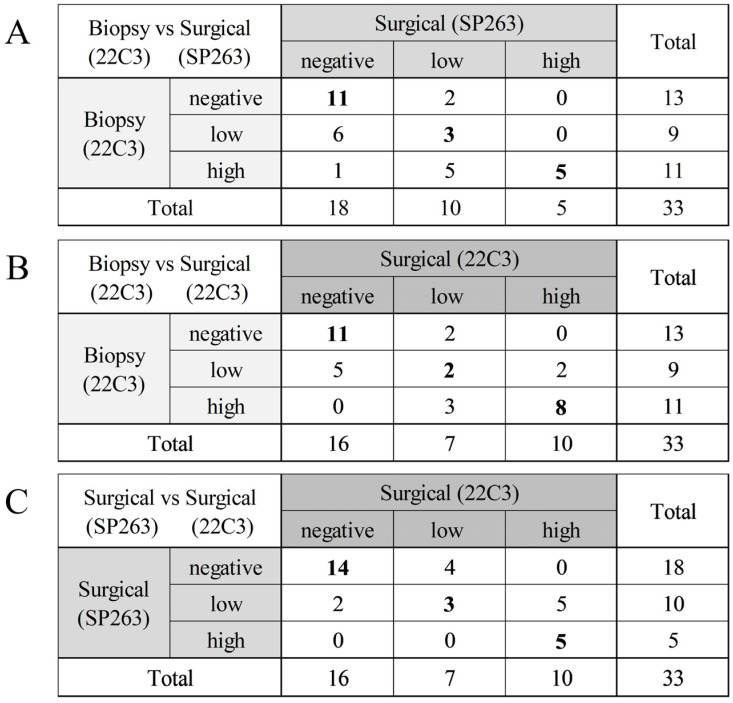
Comparison of PD-L1 expressions with 3 categorical classifications. (**A**) Between the 22C3 of the preoperative biopsy specimens and the SP263 of the surgical specimens. (**B**) Between the 22C3 of the preoperative biopsy specimens and the 22C3 the surgical specimens. (**C**) Between the 22C3 of the surgical specimens and the SP263 of the surgical specimens. Bold indicates concordant cases.

**Figure 2:**
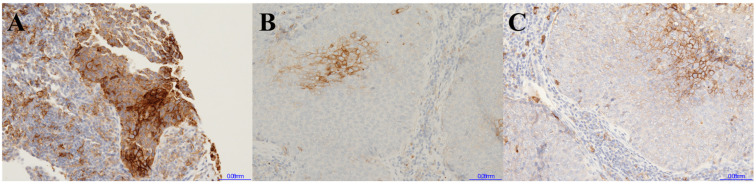
An example of IHC images of a discordant case of PD-L1 expression between the preoperative biopsy and the surgical specimen. (original magnification ×200). (**A**) The 22C3 PD-L1 expression of the preoperative computed tomography-guided percutaneous needle biopsy specimen; 90%. (**B**) The SP263 PD-L1 expression of the surgical specimens; 5–10%. (**C**) The 22C3 PD-L1 expression of the surgical specimens; 10–20%.

The concordance of the three categorical classifications of PD-L1 expression among the three groups are summarized in Figure 1 and Table 2. Using three categorical classifications, the concordance rate between the 22C3 of the preoperative biopsy specimens and the SP263 of the surgical specimens was 57.6% (Cohen’s κ score 0.349), that of the 22C3 between the preoperative biopsy specimen and the surgical specimen was 63.6% (Cohen’s κ score 0.441), and that between the 22C3 and the SP263 of the surgical specimens was 66.7% (Cohen’s κ score 0.467). Using two categorical classifications with the ≥1% cutoff, the concordance rate between the 22C3 of the preoperative biopsy specimens and the SP263 of the surgical specimens was 72.7% (Cohen’s κ score 0.465), that of the 22C3 between the preoperative biopsy specimens and the surgical specimens was 78.8% (Cohen’s κ score 0.573), and that between the 22C3 and the SP263 of the surgical specimens was 81.8% (Cohen’s κ score 0.637). Using two categorical classifications with the ≥50% cutoff, the concordance rate between the 22C3 of the preoperative biopsy specimens and the SP263 of the surgical specimens was 81.8% (Cohen’s κ score 0.526), that of the 22C3 between the preoperative biopsy specimens and the surgical specimens was 84.8% (Cohen’s κ score 0.651), and that between the 22C3 and the SP263 of the surgical specimens was 84.8% (Cohen’s κ score 0.582). A case of discordant PD-L1 category between preoperative biopsy and surgical specimen is shown in Figure 2. The concordance rates using three categorical classifications according to histology are shown in Supplemental Figure S1.

## 4. Discussion

In this study, the concordance of the three categorical classifications of PD-L1 expression between the 22C3 of preoperative biopsy specimens and the SP263 of surgical specimens was not high and the Cohen’s κ score indicated weak agreement. In addition, the concordance of the 22C3 between the preoperative biopsy specimens and the surgical specimens was also not high with similar Cohen’s κ score. When we analyzed the two cutoff values (≥1% and ≥50%), the concordance of both categorical classifications between the 22C3 of preoperative biopsy specimens and the SP263 of surgical specimens became better; however, the Cohen’s κ scores continued to indicate weak agreement. Similarly, when we analyzed with the two cutoff values, the concordance of the 22C3 between the preoperative biopsy specimens and the surgical specimens became better; however, the Cohen’s κ scores continued to indicate weak to moderate agreement.

The assessment of PD-L1 protein expression on tumor cells has limited power for selecting patients as a biomarker due to intra-tumoral heterogeneity, inter-tumoral heterogeneity, differences in PD-L1 staining, and the impact of treatment [17]. In this study, we excluded the cases who received neoadjuvant therapy, and primary lesions in all patients but one were compared between the preoperative biopsy specimens and surgical specimens; therefore, the factors of inter-tumoral heterogeneity and the impact of treatment would not contribute to our low concordance results. For differences in PD-L1 staining, the Blueprint project phase 2 study confirmed the comparability between the 22C3 and the SP263 in the serial sections of each specimen, not only for surgical specimens but also biopsy specimens, although the results showed that SP263 had a slightly higher frequency of positivity than 22C3 [13]. The study also demonstrated that among a large group of pulmonary pathologists, the overall reliability or interobserver agreement in scoring PD-L1 was very strong, although the scoring of immune cell (IC) PD-L1 staining levels remains challenging despite group training, with poor κ scores for all IC groups. Other studies also showed good concordance between SP263 and 22C3 in the serial sections of each specimen [18,19,20,21,22], although several studies have shown relatively high rates of discordant results between the SP263 and 22C3 assays [23,24,25]. Although the precise reason for the discrepancies in those studies is unclear, various possible explanations, such as tumor heterogeneity, the number of TCs in the specimen, variability in the performance of the staining platform, and variability in the performance of the observer would be considered [20]. Given the above points, the primary cause of the low concordance in PD-L1 expression between preoperative biopsy specimens and surgical specimens in our study is considered due to the intra-tumoral heterogeneity. A meta-analysis showed there was a significant difference between biopsy specimens and surgical resection specimens when the cutoff is 50% (RR = 0.69, 95% CI: 0.58–0.83, *p* < 0.01), although there was no significant difference in the detection rate of PD-L1 at the 1% cutoff between the two groups (RR = 0.89, 95% CI: 0.70–1.12, *p* = 0.33) [26]. Focusing on histological specimens for biopsy specimen types, the study showed a significant difference between biopsy and surgical resection specimens at both the 1% cut-off (RR = 0.75, 95% CI: 0.67–0.84, *p* < 0.01) and the 50% cut-off (RR = 0.64, 95% CI: 0.51–0.80, *p* < 0.01). One study demonstrated substantial inconsistencies for the percentages of cells staining positive for PD-L1 among the different small biopsy tissue microarray cores in many cases of both adenocarcinoma and squamous cell carcinoma due to geographically heterogeneous PD-L1 expression within the tumors [27].

One of the main causes of discordance in PD-L1 expression between SP263 and 22C3 of the surgical specimens in our study could be also attributed to intra-tumoral heterogeneity. This is because some discordant cases were evaluated using different cross-sections, while others were assessed from different positions within similar cross-sections between the two tests. This would be due to the fact that the 22C3 analysis on surgical specimens in this study was performed as an additional analysis; therefore, the PD-L1 evaluation was conducted later. The fading of PD-L1 expression with the age of the tumor block is known and it is especially significant when samples are older than 3 years [28,29]. Contrary to reports that PD-L1 expression decreases with specimen age, the PD-L1 expression category for 22C3 in surgical specimens was slightly higher than that for SP263, despite the later evaluation of 22C3. Therefore, the time difference did not seem to have a significant effect on PD-L1 expression in our study.

As mentioned above, the intra-tumoral heterogeneity is especially evident in various studies comparing the PD-L1 expression in biopsies with surgical resections, which could lead to inaccurate or variable scoring for PD-L1 expression with a single biopsy sample. However, the PD-L1 expression in TCs of biopsy specimens has evolved alongside advances in ICI therapy as a meaningful biomarker for predicting the efficacy of PD-1/PD-L1 inhibitors in advanced NSCLC despite of its limitations. With the advancement of immunotherapies in the perioperative treatment of NSCLC, immunotherapies have been approved for use as neoadjuvant, adjuvant, and perioperative therapy. Consequently, we are now required to decide whether to initiate immunotherapy from the preoperative stage based on the results of biopsy specimens. As a biomarker of immunotherapy, an underestimation of PD-L1 expression in biopsy specimens could potentially result in missed opportunities for effective treatment, while overestimation might increase the likelihood of imposing unnecessary preoperative treatment burdens. Preoperative immunotherapy plus chemotherapy carries the risk that approximately 20% of patients may not be able to undergo curative surgery [9,10,30,31,32]; therefore, the choice should be considered with caution. The results of trials directly comparing the efficacy and safety of neoadjuvant or perioperative immunotherapy in combination with chemotherapy versus adjuvant immunotherapy, based on PD-L1 expression in preoperative biopsy specimens, are eagerly awaited.

There were several limitations to this study. First, this study was a single-center retrospective study with a small sample size, which may limit the generalizability of the findings; therefore, further evaluation with a larger cohort would be desirable. Second, our studies evaluated almost all primary lung regions between preoperative biopsy specimens and surgical specimens. Therefore, there is a lack of comparison of PD-L1 expression in metastatic lymph nodes. Third, since this study could not evaluate the actual clinical efficacy of immunotherapies, the impacts of the disagreement of PD-L1 expression on clinical treatment are unknown. In the future, a multi-center study with a large population will be required to compare PD-L1 expression between preoperative biopsy samples and surgical specimens, including not only primary lung lesions but also hilar and mediastinal metastatic lymph nodes. Such a study should also evaluate the impact of discordant cases on the clinical course, as it would provide valuable additional insights.

## 5. Conclusions

The categorization of PD-L1 expression may be discordant between preoperative biopsy specimens and surgical specimens. PD-L1 expression evaluated with small biopsy specimens may be largely influenced by chance due to intra-tumoral heterogeneity. Therefore, careful consideration is required when using biopsy specimens from patients with resectable-stage NSCLC to assess PD-L1 status for eligibility for immunotherapy.

## Figures and Tables

**Table 1:** Patient and tumor characteristics.

	*n* = 33	(%)
Median age [range]	74	[47–89]
Gender (female)	11	33%
Smoking history		
Never	8	24%
Former/current	25	76%
Histology		
Adenocarcinoma	27	82%
Squamous cell carcinoma	6	18%
pStage (UICC 8th)		
IIA-IIB	18	55%
IIIA-IIIB	15	45%
Biopsy methods		
TBB (primary lesions)	28	85%
CTNB (primary lesions)	4	12%
EBUS-TBNA (lymph node)	1	3%
Surgical methods		
Lobectomy (primary lesions)	33	100%
Time interval from biopsy to surgery (day) [range]	35	[14–137]

Abbreviations: UICC, Union for International Cancer Control; TBB, transbronchial biopsy; CTNB, computed tomography-guided needle biopsy; EBUS-TBNA, endobronchial ultrasound-guided transbronchial needle aspiration.

**Table 2:** Concordance rates of PD-L1 expression.

Three categorical classifications		Concordance rate	Cohen’s κ score
Biopsy specimens (22C3)	Surgical specimens (SP263)	57.6	0.349 *
Biopsy specimens (22C3)	Surgical specimens (22C3)	63.6	0.441 *
Surgical specimens (SP263)	Surgical specimens (22C3)	66.7	0.467 *
Two categorical classifications with PD-L1 1% cutoff		Concordance rate	Cohen’s κ
Biopsy specimens (22C3)	Surgical specimens (SP263)	72.7	0.465 *
Biopsy specimens (22C3)	Surgical specimens (22C3)	78.8	0.573 *
Surgical specimens (SP263)	Surgical specimens (22C3)	81.8	0.637 *
Two categorical classifications with PD-L1 50% cutoff		Concordance rate	Cohen’s κ
Biopsy specimen(22C3)	Surgical specimens (SP263)	81.8	0.526 *
Biopsy specimen(22C3)	Surgical specimens (22C3)	84.8	0.651 *
Surgical specimens (SP263)	Surgical specimens (22C3)	84.8	0.582 *

Abbreviations: PD-L1, programmed death ligand-1. * Denotes statistical significance (*p*-value < 0.01).

## Data Availability

The data that support the findings of this study are available from the corresponding author upon reasonable request.

## Supplementary Materials

The following supporting information can be downloaded at: https://www.mdpi.com/article/10.3390/cancers17030398/s1, Figure S1: Comparison of PD-L1 expressions with 3 categorical classifications according to histology.
