# Supplementary material for: Comparison of PD-L1 Expression Between Preoperative Biopsy Specimens and Surgical Specimens in Non-Small Cell Lung Cancer
Source: Cancers (Basel). 2025 Jan 25;17(3):398. doi: 10.3390/cancers17030398 (PMC11815912; doi:10.3390/cancers17030398)
Supplement: Supplementary file 1 [file cancers-17-00398-s001.zip › cancers-3399021-supplementary.pdf]

Supplemental Figure S1. Comparison of PD-L1 expressions with 3 categorical classifications according to histology.

a

Biopsy vs Surgical (22C3) (SP263)		Surgical (SP263)			Total
		negative	low	high	
Biopsy (22C3)	negative	**10**	2	0	12
	low	5	**2**	0	7
	high	1	3	**4**	8
Total		16	7	4	27
Concordance: 59.2% (16/27) Cohen's κ score 0.349					

b

Biopsy vs Surgical (22C3) (22C3)		Surgical (22C3)			Total
		negative	low	high	
Biopsy (22C3)	negative	**10**	2	0	12
	low	4	**2**	1	7
	high	0	3	**5**	8
Total		14	7	6	27
Concordance: 63.0% (17/27) Cohen's κ score 0.418					

c

Surgical vs Surgical (SP263) (22C3)		Surgical (22C3)			Total
		negative	low	high	
Surgical (SP263)	negative	**12**	4	0	16
	low	2	**3**	2	7
	high	0	0	**4**	4
Total		14	7	6	27
Concordance: 70.3% (19/27) Cohen's κ score 0.500					

d

Biopsy vs Surgical (22C3) (SP263)		Surgical (SP263)			Total
		negative	low	high	
Biopsy (22C3)	negative	**1**	0	0	1
	low	1	**1**	0	2
	high	0	2	**1**	3
Total		2	3	1	6
Concordance: 50.0% (3/6) Cohen's κ score 0.280					

e

Biopsy vs Surgical (22C3) (22C3)		Surgical (22C3)			Total
		negative	low	high	
Biopsy (22C3)	negative	**1**	0	0	1
	low	1	**0**	1	2
	high	0	0	**3**	3
Total		2	0	4	6
Concordance: 66.7% (4/6) Cohen's κ score 0.455					

f

Surgical vs Surgical (SP263) (22C3)		Surgical (22C3)			Total
		negative	low	high	
Surgical (SP263)	negative	**2**	0	0	2
	low	0	**0**	3	3
	high	0	0	**1**	1
Total		2	0	4	6
Concordance: 50.0% (3/6) Cohen's κ score 0.357					

(a) Between the 22C3 of the preoperative biopsy specimens and the SP263 of the surgical specimens in adenocarcinoma. (b) Between the 22C3 of the preoperative biopsy specimens and the 22C3 of the surgical specimens in adenocarcinoma. (c) Between the 22C3 of the surgical specimens and the SP263 of the surgical specimens in adenocarcinoma. (d) Between the 22C3 of the preoperative biopsy specimens and the SP263 of the surgical specimens in squamous cell carcinoma. (e) Between the 22C3 of the preoperative biopsy specimens and the 22C3 of the surgical specimens in squamous cell carcinoma. (f) Between the 22C3 of the surgical specimens and the SP263 of the surgical specimens in squamous cell carcinoma. Bold indicates concordant cases.
